# Planning process under the National Rural Health Mission: achievements and challenges for better implementation

**DOI:** 10.1186/1753-6561-6-S5-O7

**Published:** 2012-09-28

**Authors:** Jhimly Baruah, Ritu Priya, Anuradha Jain

**Affiliations:** 1 National Health System Resource Centre, Delhi, India; 2 Jawaharlal Nehru University, Delhi, India

## Introduction

The National Rural Health Mission (NRHM) framework for implementation envisaged that District Health Action Plans would be the central hub for decentralised planning, inter-sectoral convergence, implementation and monitoring. These would feed into the annual State Programme Implementation Plans (PIP). Communitisation, including village planning, and locally suited innovations were to be encouraged. District Planning did not however emerge as a creative tool in the eleventh plan period of the Government of India.

This paper attempts to document the processes of planning under the NRHM at state, district and village levels. It also dwells on probable reasons for failure of use of the plans for resource allocation and implementation of the component strategies and activities in the plans.

## Methods

The Program Implementation Plans from 29 states, district and block plans from 20 states, and village plans from12 states were analysed over the period 2007-2011. Primary data for 31 selected indicators were collected by field visits for observation and discussion with key stakeholders. The approach adopted for planning, use of evidence for planning, and effect of capacity building of district planning teams on planning processes were analysed. Trend analysis of change in the planning teams and its effect on the plan quality, proposed strategies and utilisation was explored.

## Results

We observed major improvement in the quality of plans and processes over the early years, from 2007-2010, though with great variability across states. Different teams of state officers and external consultants did the planning in the various states, with varying outcomes in terms of ownership of plans, accountability and creation of sustainable internal capacities. Variable approaches were adopted for village and block level planning.

The use of districts plans was also variable. In most of the states, funds were allocated according to district plans. A fragmented approach to planning, funding, implementation and monitoring was observed. Consistently, strategies of convergence for the vertical disease control programmes and for action on social determinants of health remained the weak areas in the planning process.

## Discussion

The analysis clearly brings out the fact that there was improvement over the years in the situational analysis done for planning. However, this does not translate into more context specific plans. The plans made do not get implemented for several reasons. Both to ensure capacities of planning and to bring continuity in planning and implementation cycles, a public health cadre would be meaningful. It would have experts who would take health plans through a cycle of formation, funding, implementation and monitoring every year. The need thus is strengthening, expanding and capacity enhancement of public health institutions to create interdisciplinary teams who can be part of this public health cadre. In addition, the uses of health plans at various levels need to be conceptualised more realistically and creatively. The periodicity of plan preparation needs review for meaningful planning and implementation.

## Competing interests

None declared.

## Funding statement

The study was funded by the National Health Systems Resource Centre, New Delhi.

